# Triplet Chemotherapy with Cisplatin versus Oxaliplatin in the CRITICS Trial: Treatment Compliance, Toxicity, Outcomes and Quality of Life in Patients with Resectable Gastric Cancer

**DOI:** 10.3390/cancers14122963

**Published:** 2022-06-15

**Authors:** Astrid E. Slagter, Irene A. Caspers, Nicole C. T. van Grieken, Iris Walraven, Pehr Lind, Elma Meershoek-Klein Kranenbarg, Cecile Grootscholten, Marianne Nordsmark, Johanna W. van Sandick, Karolina Sikorska, Cornelis J. H. van de Velde, Edwin P. M. Jansen, Marcel Verheij, Hanneke W. M. van Laarhoven, Annemieke Cats

**Affiliations:** 1 Department of Radiation Oncology, Antoni van Leeuwenhoek/Netherlands Cancer Institute, 1066 CX Amsterdam, The Netherlands; a.slagter@nki.nl (A.E.S.); iris.walraven@radboudumc.nl (I.W.); epm.jansen@nki.nl (E.P.M.J.); marcel.verheij@radboudumc.nl (M.V.); 2 Department of Gastrointestinal Oncology, Antoni van Leeuwenhoek/Netherlands Cancer Institute, 1066 CX Amsterdam, The Netherlands; i.caspers@nki.nl (I.A.C.); c.grootscholten@nki.nl (C.G.); 3 Department of Pathology, Cancer Center Amsterdam, Amsterdam University Medical Centers, 1081 HV Amsterdam, The Netherlands; nct.vangrieken@amsterdamumc.nl; 4 Department of Epidemiology, Radboud University Medical Center, 6525 GA Nijmegen, The Netherlands; 5 Department of Oncology, Stockholm Söder Hospital, 118 83 Stockholm, Sweden; pehr.lind@ki.se; 6 Karolinska Institutet, Research Oncology, 171 77 Stockholm, Sweden; 7 Department of Surgery, Leiden University Medical Center, 2333 ZA Leiden, The Netherlands; w.m.meershoek-klein_kranenbarg@lumc.nl (E.M.-K.K.); c.j.h.van_de_velde@lumc.nl (C.J.H.v.d.V.); 8 Department of Oncology, Aarhus University, 8000 Aarhus, Denmark; marianne.nordsmark@rm.dk; 9 Department of Surgery, Antoni van Leeuwenhoek/Netherlands Cancer Institute, 1066 CX Amsterdam, The Netherlands; j.v.sandick@nki.nl; 10 Department of Biometrics, Antoni van Leeuwenhoek/Netherlands Cancer Institute, 1066 CX Amsterdam, The Netherlands; k.sikorska@nki.nl; 11 Department of Radiation Oncology, Radboud University Medical Center, 6525 GA Nijmegen, The Netherlands; 12 Department of Medical Oncology, Cancer Center Amsterdam, Amsterdam University Medical Centers, University of Amsterdam, 1081 HV Amsterdam, The Netherlands; h.vanlaarhoven@amsterdamumc.nl

**Keywords:** resectable gastric cancer, chemotherapy, cisplatin, oxaliplatin

## Abstract

**Simple Summary:**

Perioperative chemotherapy is the current standard treatment for patients with resectable gastric cancer. Either cisplatin or oxaliplatin could be part of the chemotherapy regimen, of which oxaliplatin is currently most used in the standard treatment. Evidence to choose oxaliplatin over cisplatin in the curative setting is limited. In this study, we compared cisplatin versus oxaliplatin in patients with resectable gastric cancer treated with pre- and postoperative chemotherapy. Adverse events were not different for patients who received cisplatin versus those who received oxaliplatin, nor was compliance with the treatment regimen. We could not detect survival differences between patients treated with cisplatin versus oxaliplatin. Diarrhea more frequently impacted patients treated with oxaliplatin than patients treated with cisplatin. As hydration is not needed for oxaliplatin, it is more practical to use in daily care. In conclusion, both cisplatin and oxaliplatin are legitimate options as part of systemic treatment in patients with resectable gastric cancer.

**Abstract:**

(1) Background: Perioperative chemotherapy is the current standard treatment for patients with resectable gastric cancer. Based on studies in patients with metastatic gastric cancer, oxaliplatin has replaced cisplatin in the curative setting as well. However, evidence to prefer oxaliplatin over cisplatin in the curative setting is limited. (2) Methods: We compared patient-related and tumor-related outcomes for cisplatin versus oxaliplatin in patients with resectable gastric cancer treated with perioperative chemotherapy in the CRITICS trial. (3) Results: Preoperatively, 632 patients received cisplatin and 149 patients received oxaliplatin. Preoperative severe toxicity was encountered in 422 (67%) patients who received cisplatin versus 89 (60%) patients who received oxaliplatin (*p* = 0.105). Severe neuropathy was observed in 5 (1%) versus 6 (4%; *p* = 0.009) patients, respectively. Postoperative severe toxicity occurred in 109 (60%) versus 26 (51%) (*p* = 0.266) patients; severe neuropathy in 2 (1%) versus 2 (4%; *p* = 0.209) for patients who received cisplatin or oxaliplatin, respectively. Diarrhea impacted the quality of life more frequently in patients who received oxaliplatin compared to cisplatin. Complete or near-complete pathological response was achieved in 94 (21%) versus 16 (15%; *p* = 0.126) patients who received cisplatin or oxaliplatin, respectively. Overall survival was not significantly different in both groups (*p* = 0.300). (4) Conclusions: Both cisplatin and oxaliplatin are legitimate options as part of systemic treatment in patients with resectable gastric cancer.

## 1. Introduction

Gastric cancer is the fifth most common malignancy and has a high mortality rate [1]. To improve outcomes for patients with resectable disease, several (neo-) adjuvant treatment strategies have been evaluated [2], including postoperative chemoradiotherapy [3], perioperative chemotherapy [4,5], and postoperative chemotherapy [6,7].

Publication of the MAGIC trial [4] initiated the implementation of perioperative chemotherapy as standard therapy for patients with resectable gastric cancer in Europe and the United States [8]. In the MAGIC trial, perioperative chemotherapy with epirubicin, cisplatin, and fluorouracil (5-FU; ECF) showed a survival benefit over surgery alone [4]. Shortly after, the REAL-2 trial was established in a 2 × 2 factorial design that in patients with metastasized esophagogastric cancer, capecitabine and oxaliplatin were associated with similar overall survival (OS) compared to fluorouracil and cisplatin, respectively [9]. Cisplatin was associated with higher incidences of severe neutropenia, renal toxicity, and thromboembolism, while oxaliplatin was associated with higher incidences of severe diarrhea and neuropathy. Especially the ease of administration was decisive in incorporating oxaliplatin in clinical practice as no hydration is needed for oxaliplatin while this is necessary for cisplatin [10]. Furthermore, oral capecitabine eliminated the need for a central venous access port, which was a prerequisite for infusional 5-FU. More recently, the FLOT-4 AIO study changed the landscape of perioperative treatment [5]. This trial showed better OS for patients with resectable gastric cancer treated with perioperative 5-FU, leucovorin, oxaliplatin, and docetaxel (FLOT) compared to perioperative epirubicin, cisplatin, and capecitabine/5-FU (ECX/ECF). Based on these results, FLOT has recently been implemented as standard perioperative chemotherapy in Europe and the United States, simultaneously reintroducing the need for a central venous access port due to the local painful infusion reaction by oxaliplatin and administration of infusional 5-FU.

Thus, oxaliplatin has replaced cisplatin in the perioperative setting. However, direct comparative evidence to choose oxaliplatin over cisplatin in the curative setting is lacking. This is important for establishing the optimal chemotherapy regimen in future studies. To provide input for this consideration, we made a direct comparison of patient-related and tumor-related outcomes for cisplatin and oxaliplatin in patients with resectable gastric cancer treated with perioperative chemotherapy.

## 2. Materials and Methods

Patients who were included in the CRITICS trial (ChemoRadiotherapy after Induction chemotherapy In Cancer of the Stomach) and started preoperative chemotherapy were eligible for this post-hoc analysis. In the CRITICS study, patients with resectable gastric or gastroesophageal junction (GEJ) cancer were 1:1 randomized between either perioperative chemotherapy (arm 1) or preoperative chemotherapy plus postoperative chemoradiotherapy (arm 2). The CRITICS trial recruited patients in The Netherlands, Sweden, and Denmark. The CRITICS study protocol and main results of the study have been published previously [11,12]. Both pre- and postoperative (in arm 1) chemotherapy consisted of three 3-weekly cycles of epirubicin (day 1; 50 mg/m^2^), cisplatin (day 1; 60 mg/m^2^) or oxaliplatin (day 1; 130 mg/m^2^), and capecitabine (1000 mg/m^2^ twice daily during 14 days in combination with epirubicin and cisplatin/625 mg/m^2^ twice daily during 21 days in combination with epirubicin and oxaliplatin; ECX/EOX). Administration of both cisplatin and oxaliplatin was allowed in the CRITICS trial. In The Netherlands, the use of cisplatin was preferred because oxaliplatin was not reimbursed at the start of the study. In Sweden, the use of oxaliplatin was preferred.

According to the CRITICS study protocol, dose reductions of chemotherapy were allowed, with a maximum of 50% for capecitabine in case of persisting grade 3–4 adverse events (AE). Cisplatin or oxaliplatin was discontinued in case of significant nephrotoxicity, ototoxicity, or sensory neurotoxicity. Toxicities were assessed according to the National Cancer Institute Common Toxicity Criteria (CTCAE; version 3.0). Preoperative toxicity was recorded from the administration of the first chemotherapy cycle until the administration of the last preoperative cycle plus 30 days, or surgery, which one occurred first. Postoperative toxicity was recorded from the administration of postoperative treatment until the administration of the last postoperative treatment plus 30 days. Toxicities were divided into five main groups: hematological, gastrointestinal, vascular, constitutional, and others. Individual toxicities of specific interest included diarrhea, neuropathy, renal toxicity, neutropenia, and thrombocytopenia.

In the current post-hoc analysis of the CRITICS trial, we focused on treatment compliance, toxicity, outcomes, and quality of life for patients treated with cisplatin versus oxaliplatin-containing triplet chemotherapy. Baseline demographics were compared to detect potential imbalances (preoperative and postoperative chemotherapy separately) for patients who received ECX versus EOX and included: age, gender, WHO performance status, histological subtype (Lauren classification, centrally revised), tumor localization, and allocated treatment. We compared toxicity and compliance for patients who received ECX or EOX, including relative dose intensities (RDI’s) and completion of three cycles of chemotherapy. For those patients who underwent potentially curative surgery, we evaluated the type of resection and centrally revised histopathological response according to Mandard for patients who received preoperative ECX versus patients who received preoperative EOX [13]. A good histopathological response was defined as tumor regression grade (TRG) 1 or 2 according to Mandard and a poor response was defined as TRG 3–5. For patients allocated to perioperative chemotherapy in the CRITICS trial (arm 1), we also compared postoperative toxicity and compliance for patients who received ECX versus EOX. Overall-survival estimates were evaluated for patients who received ECX versus EOX in arm 1 of the CRITICS trial. Patients who switched from ECX to EOX or vice versa during treatment were excluded from survival analyses.

In the CRITICS trial, patients were asked to complete health-related quality of life (HRQOL) questionnaires at several time points: at baseline, after preoperative therapy, after surgery, after postoperative treatment and one year after inclusion. The European Organization for Research and Treatment of Cancer Quality of Life Questionnaire-Core 30 (EORTC QLQ-C30) and the gastric cancer-specific module (EORTC QLQ-STO22) were used [14,15]. The QLQ-C30 questionnaires contain five functioning scales (physical, role, cognitive, emotional, and social functioning), three symptom scales (fatigue, nausea/vomiting, and pain), a global quality of life scale, and a number of single items for additional symptoms (dyspnea, insomnia, appetite loss, constipation, diarrhea, financial impact). All items were linearly converted into a 0–100 scale. For functional and global HRQOL, a higher score means better functioning, whereas, for symptom scales, a higher score means more symptoms. The summary score was calculated as the mean of the combined 13 scores (financial impact and global HRQOL were excluded). Outcomes on HRQOL in the CRITICS were reported previously [16]. In this analysis, we evaluated differences between patients treated with ECX and EOX in arm 1 of the CRITICS trial for predefined items (physical functioning, cognitive functioning, nausea/vomiting, diarrhea, and the QLQ-C30 summary score). Patients who switched from ECX to EOX or vice versa during treatment were excluded from HRQOL analyses.

Statistical analysis: Continuous variables such as RDI were displayed as the median plus interquartile range (IQR) and comparisons between groups were tested for significance using the Mann–Whitney U test. Categorical variables such as sex and tumor localization were displayed as frequencies and percentages and compared using the Chi-square test or the Fisher’s exact test in case one of the cells contained ten patients or less. Relative dose intensities were calculated (pre- and postoperative separately) as a function of dose and time:

$$\frac{\mathrm{dose}\;\mathrm{received}\;\left(\mathrm{mg}\right)}{\mathrm{dose}\;\mathrm{planned}\;\left(\mathrm{mg}\right)}\;\times \;\frac{\mathrm{time}\;\mathrm{planned}\;\left(\mathrm{days}\right)}{\mathrm{time}\;\mathrm{planned}\left(\mathrm{days}\right)+\;\mathrm{days}\;\mathrm{of}\;\mathrm{delay}}\;\times \;100\%.$$

Overall survival was defined as the time between randomization and date of death or last follow-up. The Kaplan–Meier method was used for survival plots and groups were compared using the Log-rank test. If any imbalances in baseline characteristics were detected, logistic regression analysis was used to correct histopathological response and Cox-regression analysis was used to correct survival outcomes. We considered variables to be imbalanced if there was a difference of 10% or larger within a variable between patients who received ECX versus EOX.

For HRQOL analyses, linear mixed modeling was used to evaluate potential differences between ECX versus EOX over time and per time-point. A model was made with a random intercept and an autoregressive covariance structure. Based on the maximum likelihood fits, improvement of fit of the models was evaluated. We compared both differences between groups with time as a continuous variable and time as a categorical variable to assess differences between time points. We reported differences in mean scores over time between ECX and EOX and we provided Cohen’s effect size (ES) [17]. An ES of 0.20 is considered small, an ES of 0.50 as moderate and clinically significant, and an ES of 0.80 as large [18].

Analyses were performed in SPSS version 27.0, Kaplan–Meier estimates were performed using R statistical software version 4.0.3. The threshold for statistical significance was considered *p* < 0.05.

## 3. Results

### 3.1. Baseline Characteristics

**Figure 1:**
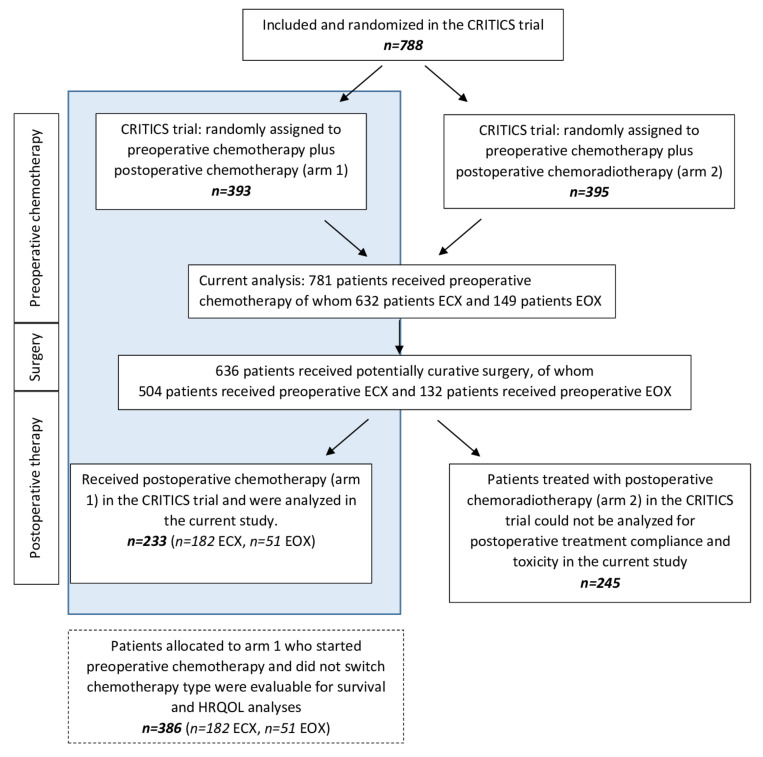
Flowchart of the study.

A total of 788 patients were included in the CRITICS trial. Among them, 781 patients started preoperative therapy and were eligible for the current analysis. Preoperatively, 632 patients received preoperative ECX and 149 patients received preoperative EOX in either arm 1 or 2 of the CRITICS trial. Figure 1 shows a flowchart of the current study.

Baseline characteristics are displayed in Table 1. A few imbalances were detected between patients who received ECX versus patients who received EOX. The subtype of gastric cancer was not equally distributed between patients who received ECX compared to patients who received EOX (*p* = 0.001). Intestinal type of cancer was more common in patients who received ECX, whereas mixed and other types were more common in patients who received EOX. In addition, the country of inclusion was highly correlated to the type of chemotherapy. Of 632 patients in the ECX group, 614 (97%) were treated in The Netherlands, while 136 out of 149 patients in the EOX group were treated in Sweden (*p* < 0.001).

Five patients switched from cisplatin to oxaliplatin during treatment; two patients switched after the second preoperative course and two postoperatively. For two patients, the reason for switch was thromboembolism. One patient switched from oxaliplatin to cisplatin after the second preoperative course. These patients were excluded for survival and HRQOL analysis.

### 3.2. Preoperative Treatment Compliance and Toxicity

Preoperatively, we did not detect major differences in RDI between patients treated with ECX versus patients treated with EOX. Relative dose intensities are shown in Table 2 and Supplementary Materials Figure S1. Completion of the third cycle of preoperative chemotherapy was not significantly different and succeeded in 524 (83%) patients treated with ECX and 131 patients (88%) treated with EOX (*p* = 0.135).

The occurrence of severe (grade 3–5) toxicity was slightly higher in the ECX group: 422 (67%) patients in the ECX group versus 89 (60%) patients in the EOX group (*p* = 0.105). To illustrate, the highest grade of any toxicity was grade 3 in 302 (48%) versus 66 (44%) patients, grade 4 in 108 (17%) versus 22 (15%) patients and grade 5 in 12 (2%) versus 1 (1%) for patients who received preoperative ECX versus EOX, respectively. More details on toxicity are given in Table 3. With respect to the specific individual toxicities of interest: severe diarrhea occurred in 78 (12%) versus 24 (16%) patients (*p* = 0.225), severe renal toxicity in 12 (2%) versus 0 (0%) patients (*p* = 0.137), severe neutropenia in 205 (32%) versus 45 (30%) patients (*p* = 0.621), severe thrombocytopenia in 56 (9%) versus 9 (6%) patients (*p* = 0.232), and severe neuropathy in 5 (1%) versus 6 (4%) patients (*p* = 0.009).

### 3.3. Postoperative Treatment Compliance and Toxicity

A total of 392 patients were allocated to postoperative chemotherapy (arm 1), of whom 233 (59%) patients actually started postoperative chemotherapy. No major differences in RDI’s were detected between patients who received postoperative ECX or postoperative EOX. Details can be found in Table 2 and Supplementary Materials Figure S2. In addition, completion of three cycles of postoperative chemotherapy succeeded in 142 (78%) patients in the ECX group versus 38 (75%) patients in the EOX group (*p* = 0.597).

Also during postoperative chemotherapy, the occurrence of severe postoperative toxicity occurred was slightly higher in the ECX group: 109 (60%) in patients treated with ECX and 26 (51%) in patients treated with EOX (*p* = 0.266). Highest grade of any postoperative toxicity was grade 3 in 91 (50%) versus 22 (43%) patients and grade 4 in 18 (10%) versus 4 (8%) patients who received postoperative ECX versus EOX, respectively. Table 3 shows more details on toxicity. No significant differences between ECX and EOX were detected in the specific individual toxicities of interest. Severe diarrhea occurred in 9 (5%) versus 4 (8%) patients (*p* = 0.489), renal toxicity in 3 (2%) versus 0 (0%) patients (*p* = 0.999), severe neutropenia in 64 (35%) versus 15 (29%) patients (*p* = 0.505), thrombocytopenia in 5 (3%) versus 0 (0%) patients (*p* = 0.588) patients and severe neuropathy in 2 (1%) versus 2 (4%) patients (*p* = 0.209) treated with postoperative ECX versus EOX, respectively.

### 3.4. Surgery and Histopathological Response

Patients treated with ECX were less likely to undergo potentially curative surgery. Potentially curative surgery was performed in 504 (80%) patients who received preoperative ECX and in 132 (87%) patients who received preoperative EOX (*p* = 0.013). In addition, the type of resection was significantly different (*p* < 0.001) between patients who received ECX versus EOX, i.e., total gastric resection was performed in 229 (45%) versus 89 (67%) of patients, subtotal gastric resection was performed in 224 (44%) versus 31 (24%) of patients and esophagus plus cardia resection was performed in 51 (10%) versus 12 (9%) of, respectively. Type of resection was highly correlated to tumor localization (*p* < 0.001, data not shown), which we used to correct outcome measures.

Evaluation of histopathological response was based on data generated during central revision and was available for 546 (87%) patients who underwent potentially curative surgery. Good histopathological response was slightly more often achieved in the ECX group, although this did not reach statistical significance: 94 (21%) for patients who received preoperative ECX compared to 16 (15%) patients who received preoperative EOX (*p* = 0.126) (OR 0.64 (95% CI 0.36–1.14) (*p* = 0.128). When we corrected the histopathological response for tumor localization and Lauren classification, the OR changed marginally (Supplementary Materials Table S1).

### 3.5. Overall-Survival

**Figure 2:**
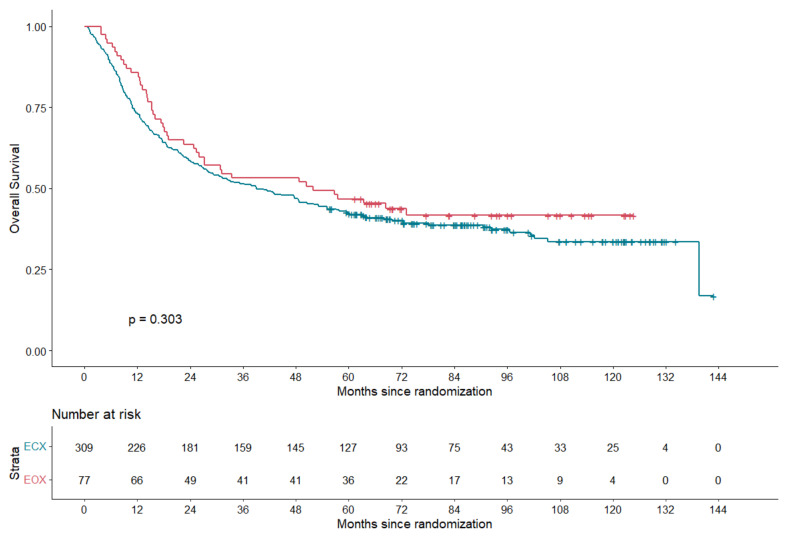
Overall-survival estimates for patients treated with ECX versus EOX. HR 0.84 (95% CI 0.61–1.17).

The median follow-up time in this study was 88 months. OS was evaluated among 386 patients: 309 patients in the ECX group and 77 patients in the EOX group. The 5-year OS rate was comparable: 42% (95% CI 37–48%) in the ECX group compared to 47% (95% CI 37–59%) in the EOX group (*p* = 0.303) (Figure 2); HR 0.84 (95% CI 0.61–1.17). After correction for tumor localization and Lauren classification, the HR was 0.75 (95% CI 0.51–1.11) (*p* = 0.146) for patients treated with EOX compared to patients treated with ECX.

### 3.6. Quality of Life

**Figure 3:**
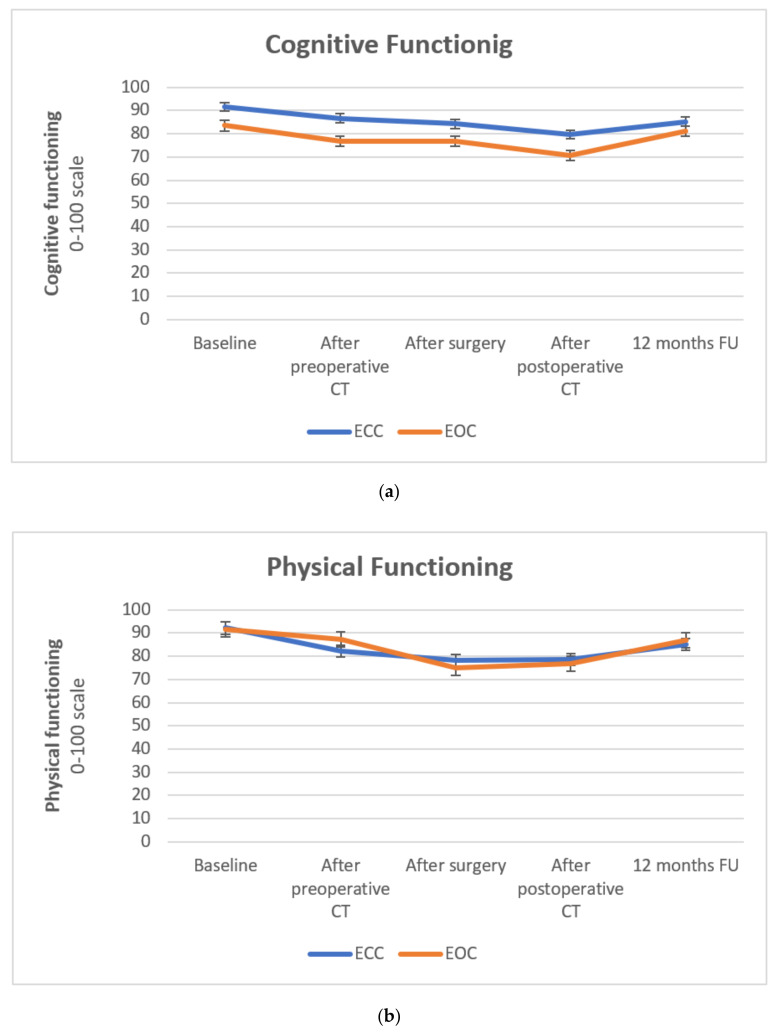

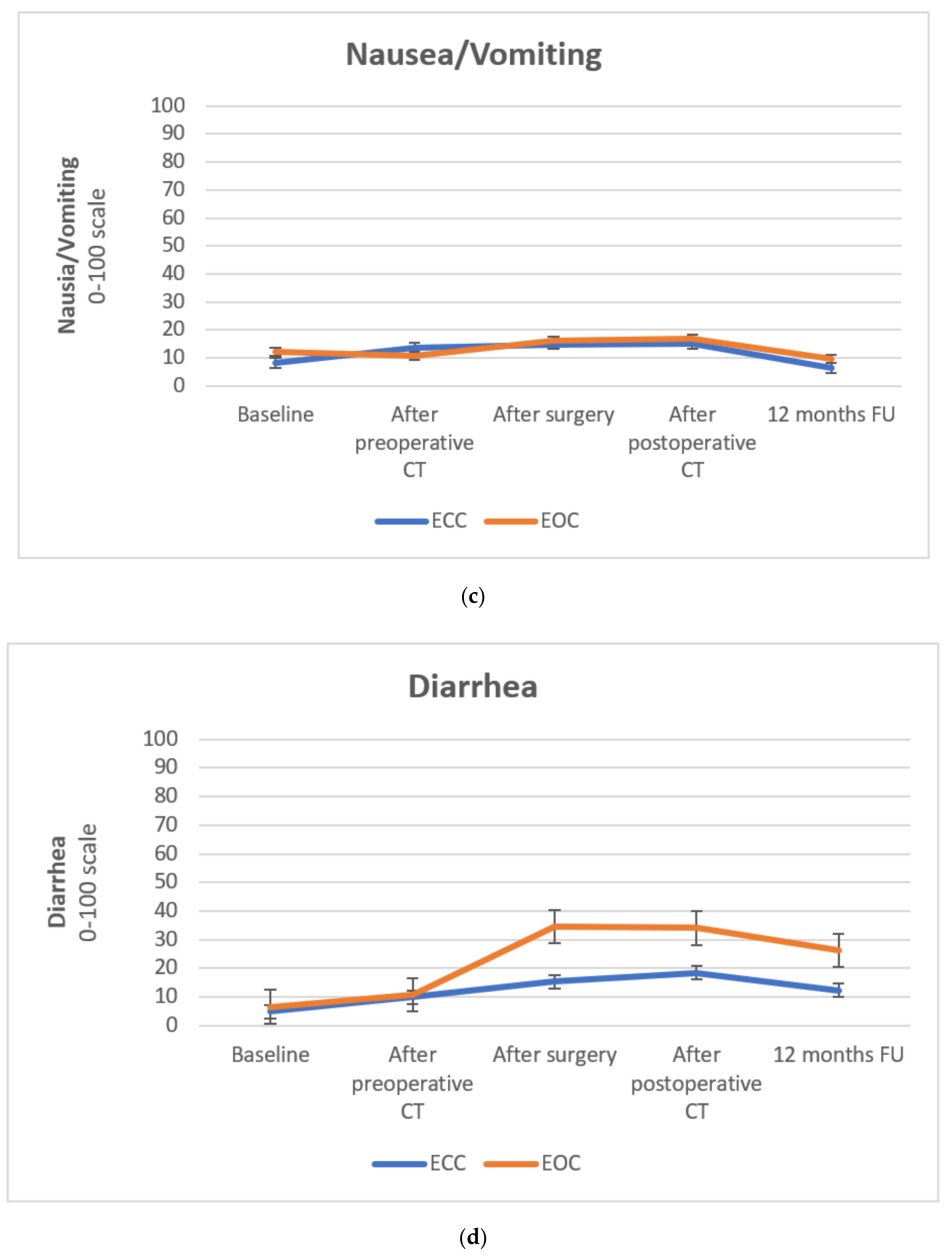

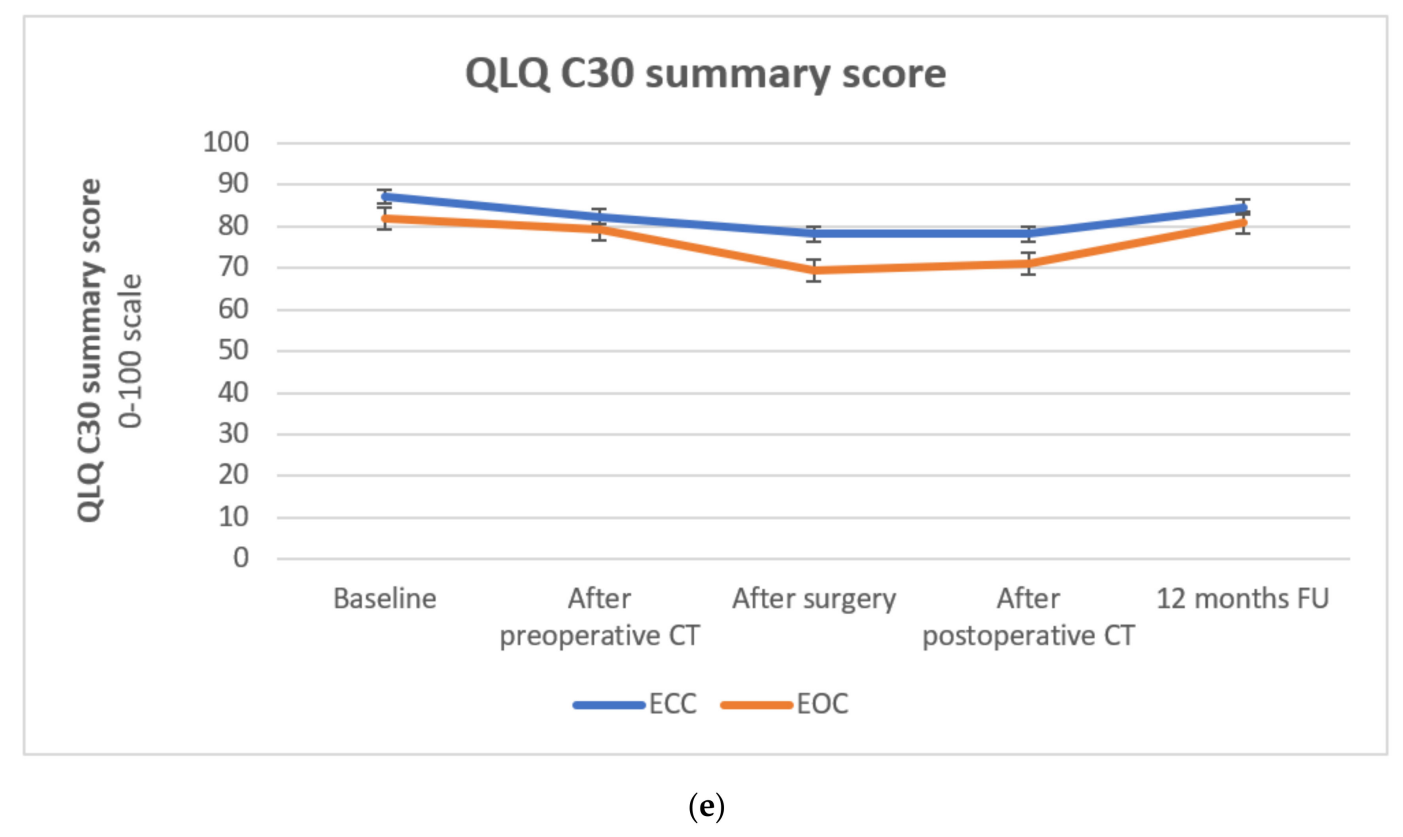
HRQOL scores at baseline, after preoperative chemotherapy, after surgery, after postoperative chemotherapy, and at 12-month follow-up (from randomization). (**a**) Cognitive functioning. A higher score means better cognitive functioning. Interaction between time and group (ECX versus EOX): *p* = 0.629, (**b**) Physical functioning. A higher score means better physical functioning. Interaction between time and group (ECX versus EOX): *p* = 0.801, (**c**) Nausea/vomiting. A higher score means more symptoms of nausea/vomiting. Interaction between time and group (ECX versus EOX): *p* = 0.950, (**d**) Diarrhea. A higher score means more symptoms of diarrhea. Interaction between time and group (ECX versus EOX): *p* = 0.544, (**e**) QLQ C30 summary score. A higher score means a better overall score. Interaction between time and group (ECX versus EOX): *p* = 0.734.

Significantly more diarrhea complaints were registered in the EOX group compared to the ECX group at each time-point following surgery (after correction for tumor localization) (Table 4, Figure 3). The mean difference in HRQOL score after surgery compared to baseline was −18.84 (standard error 3.50), favoring ECX (effect size −1.60) (*p* < 0.001) (Table 5). This difference in diarrhea burden remained significantly higher in the EOX group at one year of follow-up, with a mean difference compared to a baseline of −13.56 (standard error 3.95) and an effect size of −1.15 (*p* = 0.001). There were no significant differences between ECX versus EOX for the other investigated variables: cognitive functioning, physical functioning, nausea/vomiting, and the QLQ C30 summary score (Table 4 and Table 5, Figure 3).

## 4. Discussion

In this study, we compared patient- and tumor-related outcomes for cisplatin versus oxaliplatin in patients with resectable gastric cancer who underwent perioperative chemotherapy. Overall severe toxicity during chemotherapy treatment was not significantly different between patients who received cisplatin compared to those who received oxaliplatin. The occurrence of severe neuropathy during treatment and the burden of diarrhea following surgery and several months onwards were higher in patients who received oxaliplatin compared to patients who received cisplatin. Pathological response and survival were not significantly different between groups. To the best of our knowledge, this is the first time that a direct comparison of cisplatin and oxaliplatin combination chemotherapy has been performed in patients with gastric cancer undergoing treatment with curative intent.

The only randomized study which directly compared cisplatin with oxaliplatin in patients with gastric cancer is the REAL-2 trial, in which 1002 patients with advanced esophagogastric cancer received triplet chemotherapy with palliative intent [9]. Oxaliplatin and capecitabine were investigated as an alternative for cisplatin and fluorouracil, respectively, with epirubicin as the third agent in all groups. With respect to toxicity, the REAL-2 trial showed that oxaliplatin was associated with higher incidences of severe diarrhea and neuropathy, while cisplatin was associated with higher incidences of severe neutropenia, renal toxicity, and thromboembolism. In the current study, only neuropathy reached statistical significance and was higher in patients during oxaliplatin than in patients during cisplatin. Notwithstanding this, absolute differences in overall severe toxicity were 7–9% favoring oxaliplatin over cisplatin.

While oxaliplatin-induced neuropathy is characterized by both an acute phase, which can be triggered by cold stimulation, and a chronic phase, which develops more gradually due to nuclear and mitochondrial damage, oxidative overload stress, glia activation and neuroinflammation, cisplatin neuropathy is characterized by a chronic phase only [19]. Neurotoxicity as a side-effect of platinum can persist for a long time. In a study that included patients with colon cancer treated with adjuvant oxaliplatin-containing chemotherapy, low-grade neurotoxicity was reported in up to 20% of patients at 18 months after treatment [20]. The higher incidence of neurotoxicity for oxaliplatin compared to cisplatin is likely to be explained by platinum accumulation in the nerves, as shown in in vitro research. In platinum-treated rats, platinum retention by the dorsal root ganglia after a recovery period of 8 weeks was larger after treatment with oxaliplatin compared to cisplatin [21]. In our study, no long-term follow-up on toxicity beyond one year is available.

The burden of diarrhea, as reported in HRQOL diarrhea questionnaires, was significantly higher in the EOX group compared to the ECX group from the post-surgery time point. It is known that complaints of diarrhea occur more often in patients who underwent a total gastric resection, which was more frequently performed in the EOX group, compared to patients who underwent a subtotal gastric resection [22,23]. However, even after correction for tumor localization (which is highly correlated to the type of resection), the diarrhea burden remained significantly higher in the EOX group. And although we did not observe differences between cisplatin and oxaliplatin in severe diarrhea according to the CTCAE criteria, it is in line with results from the REAL-2 trial and a large meta-analysis [9,24] reporting higher incidences of severe diarrhea following treatment with oxaliplatin compared to cisplatin. This observation underscores the importance of patients’ reported outcomes. Diarrhea is a known acute toxicity of platinum therapy and it is also known that diarrhea can persist for years after therapy. In the case of long-term diarrhea, it is hypothesized that enteric neurons have been damaged by platinum, which causes disorders in motility and secretion [25]. In a mouse model, enteric damage on platinum therapy has been associated with length of administration [21]. This might explain why the difference in diarrhea between cisplatin and oxaliplatin was detected after a few cycles.

In line with our study, the REAL-2 trial showed slightly and non-significantly better OS for patients who received oxaliplatin compared to patients who received cisplatin [9]. A large meta-analysis that included 10,249 patients with esophagogastric cancer treated with palliative intent concluded that non-cisplatin-containing fluoropyrimidine doublets are preferred as a first-line routine treatment, of which the fluoropyrimidine and oxaliplatin or taxane combination was the most promising in terms of OS [26]. Although the chemotherapy schedule used in the current study is no longer considered standard therapy, this subgroup analysis provides important information which could be used to design future trials. The current standard treatment for patients with resectable gastric cancer is perioperative FLOT, based on the results of the FLOT-4 AIO trial [5]. Some recent studies, such as the PERTRARCA trial and the RAMSES-FLOT trial, showed that FLOT in combination with immunotherapy could substantially increase the incidence of severe toxicity in patients with gastric cancer [27,28]. Other chemotherapy backbones than FLOT could be considered in combination with immunotherapy. These backbones could include cisplatin or oxaliplatin, or maybe even other platinum compounds such as carboplatin or tetraplatin. Doublet instead of triplet chemotherapy backbone for immunotherapy could also be explored. A meta-analysis among patients with advanced HER-2 positive esophagogastric cancer revealed that in combination with trastuzumab, a doublet with oxaliplatin is preferable as first-line treatment over a doublet with cisplatin in terms of OS and was associated with less toxicity [29]. The preferable chemotherapy backbone in combination with other types of immunotherapy and in other subtypes of gastric cancer remains unknown. In addition, little is known about potential antagonistic interactions or synergistic working mechanisms of chemotherapy and immunotherapy. Preclinical studies are of great importance to enable optimal integration of immunotherapy [30].

This study has some limitations. First, it is a post-hoc analysis of the CRITICS trial, which was not designed to answer the research questions defined in the current analysis. In addition, the sample size of this study was relatively small, especially the number of patients that received oxaliplatin. Another limitation, as earlier mentioned, is that patients treated in The Netherlands and Denmark were mainly treated with ECX, while patients from Sweden were mainly treated with EOX. This could have biased the results. Finally, as earlier mentioned, toxicity beyond one year has not been recorded.

## 5. Conclusions

In conclusion, no significant survival differences were observed between resectable gastric cancer patients treated with cisplatin versus patients treated with oxaliplatin. On the one hand, the occurrence of neuropathy during treatment and diarrhea following surgery and onwards were slightly higher in the oxaliplatin group compared to the cisplatin group. On the other hand, oxaliplatin is not dependent on hydration for renal safety compared to cisplatin. In conclusion, cisplatin and oxaliplatin are both legitimate options as part of systemic treatment in patients with resectable gastric cancer and could both be considered in future trials.

## Figures and Tables

**Table 1:** Baseline characteristics for patients who started preoperative chemotherapy and for patients who started postoperative chemotherapy, respectively. Abbreviations: The Netherlands (NL), Denmark (DK), Sweden (SE).

Variable	Preoperative Chemotherapy			Postoperative Chemotherapy		
	ECX (*n* = 632)	EOX (*n* = 149)	*p*-Value	ECX (*n* = 182)	EOX (*n* = 51)	*p*-Value
Age						
Median (IQR)	62 (54–69)	62 (56–68)	0.634	60 (50–67)	62 (55–69)	0.119
Sex						
Male	423 (67%)	100 (67%)	0.966	130 (71%)	38 (74.5%)	0.665
Female	209 (33%)	49 (33%)		52 (29%)	13 (24.5%)	
WHO PS						
missing	42	2	0.916	12		0.584
0	424 (72%)	105 (71%)		128 (75%)	36 (71%)	
1	166 (28%)	42 (29%)		42 (25%)	15 (29%)	
Tumor localization						
GEJ	107 (17%)	32 (22%)	0.179	25 (14%)	9 (18%)	0.180
Proximal	120 (19%)	36 (24%)		32 (18%)	15 (29%)	
Middle	189 (30%)	39 (26%)		62 (34%)	12 (24%)	
Distal	216 (34%)	42 (28%)		63 (35%)	15 (29%)	
Lauren classification						
missing	147	27	0.001	39	14	0.042
Diffuse	211 (44%)	59 (48%)		63 (44%)	17 (46%)	
Intestinal	223 (46%)	38 (31%)		65 (46%)	11 (29%)	
Mixed	16 (3%)	12 (10%)		3 (2%)	4 (11%)	
Other	35 (7%)	13 (11%)		12 (8%)	5 (14%)	
Country of inclusion						
NL	614 (97%)	2 (1%)	<0.001	176 (97%)	6 (12%)	<0.001
DK	17 (3%)	11 (7%)		5 (3%)	1 (2%)	
SE	1 (0%)	136 (91%)		1 (0%)	44 (86%)	
Allocated treatment						
Postop CT	314 (50%)	78 (52%)	0.558			
Postop CRT	318 (50%)	71 (48%)				

**Table 2:** Relative dose intensities are displayed as median plus interquartile range.

Variable	Preoperative Chemotherapy			Postoperative Chemotherapy		
	ECX (*n* = 632)	EOX (*n* = 149)	*p*-Value	ECX (*n* = 182)	EOX (*n* = 51)	*p*-Value
Epirubicin			0.349	Missing *n* = 2		0.972
	96 (76–100)%	97 (83–100)%		83 (63–99)%	84 (62–99%)	
Cisplatin/oxaliplatin	Missing *n* = 11	Missing *n* = 1	0.572	Missing *n* = 5	Missing *n* = 1	0.117
	95 (81–100)%	96 (85–100)%		87 (65–99)%	72 (58–98)%	
Capecitabine	Missing *n* = 24	Missing *n* = 6	0.010	Missing *n* = 8	Missing *n* = 1	0.024
	91 (71–100)%	89 (72–95%)		78 (57–98)%	72 (48–85)%	

**Table 3:** Pre- and postoperative grade 3–5 toxicity.

Variable	Preoperative Chemotherapy			Postoperative Chemotherapy		
	ECX (*n* = 632)	EOX (*n* = 149)	*p*-Value	ECX (*n* = 182)	EOX (*n* = 51)	*p*-Value
Any grade 3–5	422 (67%)	89 (60%)	0.105	109 (60%)	26 (51%)	0.266
Constitutional	212 (34%)	39 (26%)	0.097	47 (26%)	8 (16%)	0.191
Gastrointestinal	158 (25%)	37 (25%)	0.999	39 (21%)	6 (12%)	0.160
Hematological	236 (37%)	46 (31%)	0.155	66 (36%)	15 (29%)	0.409
Vascular	47 (7%)	5 (3%)	0.098	4 (2%)	0 (0%)	0.579
Other	120 (19%)	29 (19%)	0.908	21 (12%)	4 (8%)	0.611
Toxicities of specific interest						
Diarrhea	78 (12%)	24 (16%)	0.225	9 (5%)	4 (8%)	0.489
Renal	12 (2%)	0 (0%)	0.137	3 (2%)	0 (0%)	0.999
Neutropenia	205 (32%)	45 (30%)	0.627	64 (35%)	15 (29%)	0.505
Thrombocytopenia	56 (9%)	9 (6%)	0.323	5 (3%)	0 (0%)	0.588
Neuropathy	5 (1%)	6 (4%)	0.009	2 (1%)	2 (4%)	0.209

***Hematological*** *included anemia, leucopenia, neutropenia, lymphocytopenia, febrile neutropenia, and thrombocytopenia. **Gastrointestinal** included mucositis/stomatitis, heartburn/dyspepsia, dysphagia, anorexia, nausea/vomiting, diarrhea, constipation, bowel inflammation, and gastrointestinal fistula/obstruction/perforation. **Vascular** included cardiac arrhythmia, ischemic event, thromboembolic event, sudden death, and hemorrhage. **Constitutional** included weight loss, dehydration, dizziness, fatigue, hypertension, infection without neutropenia, insomnia, mood alteration, and pain. **Others** included allergic/dermatological reaction, alopecia, DPD deficiency, dyspnea, genitourinary obstruction, local complication, metabolic disorder, musculoskeletal disorder, neuro-/ototoxicity, palmar-plantar erythrodysesthesia, psychosis, renal toxicity, and other toxicity.*

**Table 4:** Quality of Life items on a 0–100 scale. SE = standard error.

	T0 (Baseline)		T1 (After Preoperative CT)		T2 (After Surgery)		T3 (After Postoperative CT/CRT)		T4 (12 Months Follow Up)	
	*n*=	Mean (SE)	*n*=	Mean (SE)	*n*=	Mean (SE)	*n*=	Mean (SE)	*n*=	Mean (SE)
Cognitive Functioning										
ECX	232	91.55 (1.04)	106	86.65 (1.56)	115	84.26 (1.77)	105	79.64 (2.04)	66	85.23 (2.20)
EOX	65	83.49 (1.95)	46	76.68 (2.51)	40	76.80 (3.01)	35	70.72 (3.50)	26	81.14 (3.55)
Physical Functioning										
ECX	232	92.15 (0.80)	108	82.17 (1.37)	115	78.17 (1.65)	106	78.71 (1.75)	66	85.23 (1.46)
EOX	65	91.61 (1.51)	46	87.26 (2.19)	40	75.07 (2.84)	35	76.71 (3.04)	26	86.76 (2.38)
Nausea/Vomiting										
ECX	233	8.39 (1.08)	108	13.52 (1.81)	115	14.93 (1.92)	107	14.97 (1.88)	66	6.28 (1.42)
EOX	65	12.14 (2.04)	46	10.93 (2.82)	40	16.23 (3.31)	35	17.06 (3.29)	26	9.85 (2.29)
Diarrhea										
ECX	230	4.92 (1.01)	106	9.99 (2.26)	113	15.33 (2.47)	105	18.51 (2.61)	66	12.31 (2.53)
EOX	65	6.55 (1.91)	46	10.86 (3.45)	40	34.66 (4.18)	35	34.13 (4.51)	26	26.25 (4.10)
QLQ-C30 summary score										
ECX	226	87.08 (0.78)	105	82.25 (1.16)	110	78.10 (1.52)	104	78.11 (1.49)	65	84.58 (1.60)
EOX	63	81.78 (1.47)	45	79.31 (1.84)	40	69.37 (2.57)	35	70.95 (2.56)	26	80.79 (2.57)

**Table 5:** Quality of Life. Mean changes in mean and effect sizes. SE = standard error. ES = effect sizes.

	T0–T1 Between-Group Difference			T0–T2 Between-Group Difference			T0–T3 Between-Group Difference			T0–T4 Between-Group Difference		
	Mean Change (SE)	*p*	ES	Mean Change (SE)	*p*	ES	Mean Change (SE)	*p*	ES	Mean Change (SE)	*p*	ES
Cognitive Functioning	1.91 (2.78)	0.492	0.12	−0.61 (3.90)	0.876	−0.04	0.86 (4.36)	0.845	0.05	−3.98 (4.50)	0.379	−0.25
Physical Functioning	−5.63 (2.49)	0.025	−0.49	2.56 (3.32)	0.441	0.21	1.83 (3.57)	0.609	0.15	−2.08 (2.95)	0.482	−0.17
Nausea/Vomiting	6.34 (3.63)	0.083	0.38	2.45 (3.78)	0.518	0.15	1.66 (4.10)	0.686	0.10	0.18 (3.44)	0.959	0.01
Diarrhea	0.77 (4.48)	0.864	0.05	−17.68 (5.06)	0.001	−1.15	−13.98 (5.56)	0.013	−0.91	−12.30 (5.12)	0.018	−0.80
Corrected *	−1.13 (3.49)	0.365	−0.10	−18.84 (3.50)	<0.001	−1.60	−15.34 (3.66)	0.018	−1.30	−13.56 (3.95)	0.001	−1.15
QLQ C30 summary score	−2.36 (2.18)	0.280	−0.20	3.44 (2.95)	0.246	0.29	1.86 (3.02)	0.538	0.16	−1.50 (3.10)	0.629	−0.13

* corrected for tumor localization.

## Data Availability

Data that support the findings in this study are currently not publicly available but can be made available upon request after contact with the corresponding author.

## Supplementary Materials

The following supporting information can be downloaded at: https://www.mdpi.com/article/10.3390/cancers14122963/s1, Table S1: Multivariable logistic regression analysis with as outcome good response (Mandard 1–2) in patients who underwent surgery with curative intent; Figure S1: RDI distributions preoperatively (a) epirubicin, (b) cisplatin/oxaliplatin, (c) capecitabine; Figure S2: RDI distributions postoperatively (a) epirubicin, (b) cisplatin/oxaliplatin, (c) capecitabine.
