# Supplementary material for: Triplet Chemotherapy with Cisplatin versus Oxaliplatin in the CRITICS Trial: Treatment Compliance, Toxicity, Outcomes and Quality of Life in Patients with Resectable Gastric Cancer
Source: Cancers (Basel). 2022 Jun 15;14(12):2963. doi: 10.3390/cancers14122963 (PMC9221508; doi:10.3390/cancers14122963)
Supplement: Supplementary file 1 [file cancers-14-02963-s001.zip › cancers-1749916-supplementary.pdf]

**Supplementary Table S1:** Multivariable logistic regression analysis with as outcome good response (Mandard 1–2) in patients who underwent surgery with curative intent. Included in analysis: 345 patients with a poor response and 83 patients with a good response. * reference. GEJ = gastro-esophageal junction.

| Variable | OR for good response | 95% CI | p-value |
|---|---|---|---|
| Type of chemotherapy | | | 0.189 |
| ECX | * | | |
| EOX | 0.63 | 0.32–1.25 | |
| Lauren classification | | | |
| Intestinal | * | | |
| Diffuse | 0.44 | 0.25–0.77 | 0.004 |
| Mixed | 0.81 | 0.21–3.05 | 0.750 |
| Other | 1.04 | 0.44–2.48 | 0.931 |
| Tumor localization | | | |
| GEJ | * | | |
| Proximal | 1.12 | 0.49–2.59 | 0.790 |
| Middle | 1.02 | 0.45–2.34 | 0.956 |
| Distal | 1.36 | 0.64–2.92 | 0.426 |

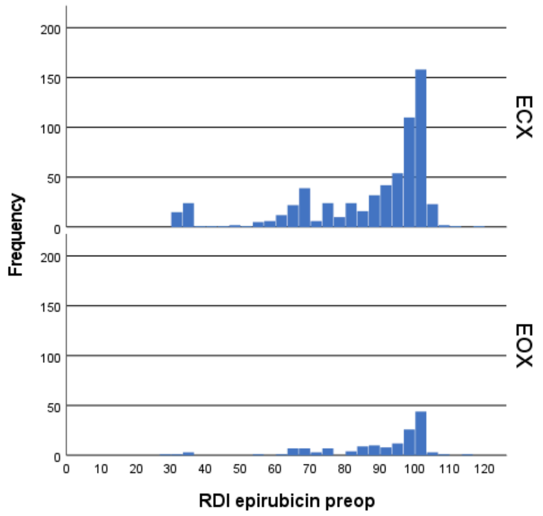


a)

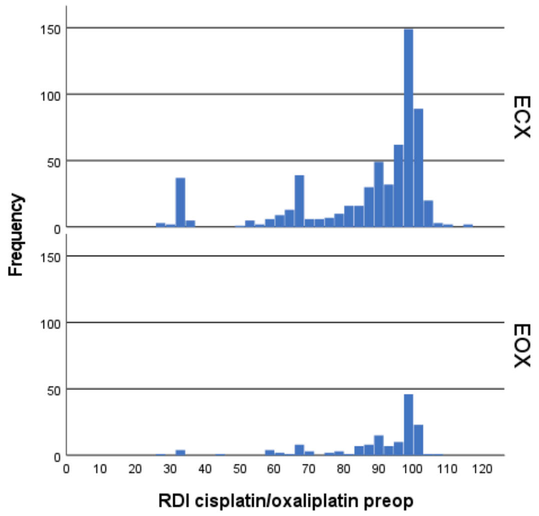


b)

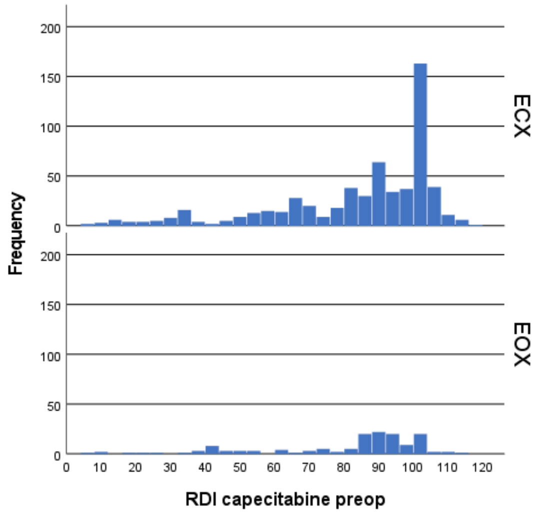


c)

**Supplementary Figure S1:** RDI distributions preoperatively a) epirubicin, b) cisplatin/oxaliplatin, c) capecitabine.

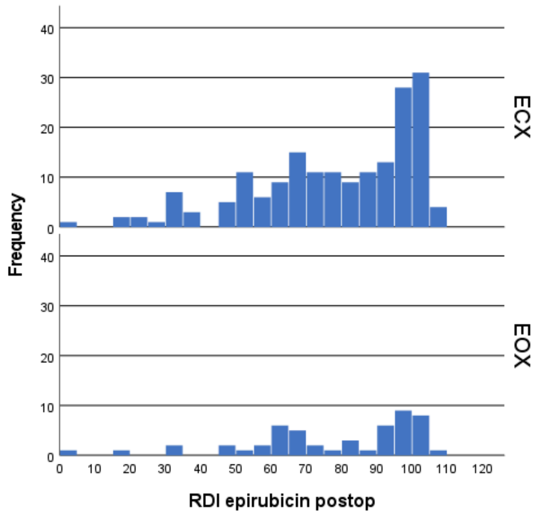


a)

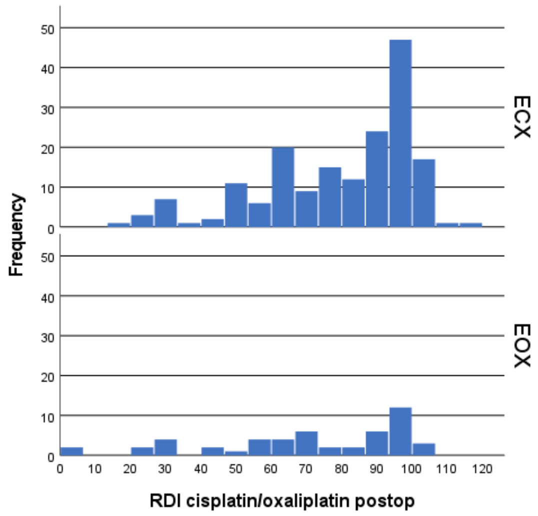


b)

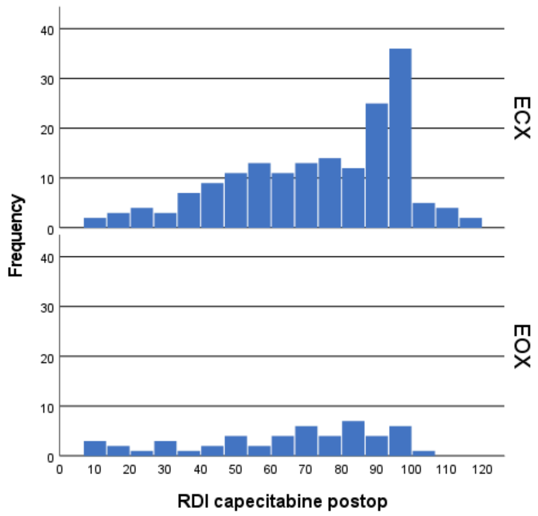


c)

**Supplementary Figure S2**: RDI distributions postoperatively a) epirubicin, b) cisplatin/oxaliplatin, c) capecitabine
